# Effectiveness of 0.1% Cyclosporine a Cationic Emulsion for Treating Dry Eye Disease After Cataract Surgery Analyzed Using a Placido Tear Film Analyzer

**DOI:** 10.3390/diagnostics15080981

**Published:** 2025-04-12

**Authors:** Song-A Che, Sang Beom Han, Yongwoo Lee

**Affiliations:** 1 Department of Ophthalmology, Kangwon National University Hospital, Kangwon National University School of Medicine, Chuncheon 24289, Republic of Korea; apple3691012@gmail.com; 2 Saevit Eye Hospital, Goyang 10447, Republic of Korea; msbhan@nate.com; 3 Department of Ophthalmology, Columbia University Irving Medical Center, Columbia University, New York, NY 10027, USA

**Keywords:** dry eye disease, cyclosporine, cationic emulsion, cataract, cyclosporine 0.1%, Placido tear film analyzer

## Abstract

**Background/Objectives**: We aimed to evaluate the effectiveness of a 0.1% cyclosporin A cationic emulsion (CsA-CE) for dry eye disease (DED) post-cataract surgery using the DED index measured with a Placido tear film analyzer. **Methods**: We retrospectively reviewed the medical records of patients who underwent simple cataract surgery. All patients used 0.5% moxifloxacin and 1% prednisolone acetate eye drops four times daily postoperatively. They were divided into the CsA-CE and control groups based on whether they had used CsA-CE 1 week after surgery. Subjective and objective assessments were performed at the baseline and 1 month postoperation. The non-invasive tear meniscus height, non-invasive tear break-up time, conjunctival redness, meibomian gland morphology, and lipid layer thickness were assessed using the Keratograph 5M (Oculus). **Results**: No differences were observed in the preoperative dry eye parameters between the groups. The ocular surface disease index decreased from 19.26 to 14.58 (*p* = 0.046) at 1 month postoperation for the CsA-CE group, and the average non-invasive tear break-up time significantly increased from 10.97 to 13.00 s (*p* = 0.002). No such differences were observed for the control group. Nasal bulbar conjunctival hyperemia increased (*p* < 0.001) for the control group. Nasal limbal hyperemia and overall limbal hyperemia increased for both groups (CsA-CE, *p* = 0.005, 0.017; control, *p* = 0.001, 0.012). The lipid layer thickness increased from 70.29 to 86.41 nm for the CsA-CE group (*p* < 0.001), whereas no significant change was noted for the control group. **Conclusions**: CsA-CE (0.1%) is effective for treating DED after cataract surgery and improves the tear lipid layer.

## 1. Introduction

Cataract surgery is being increasingly performed and has become more common globally [1,2]. Among its complications, dry eye disease (DED) is not severe but remains a concern because it significantly influences the patient satisfaction after cataract surgery [3,4]. A study reported DED as the second most common cause of dissatisfaction among patients who underwent multifocal cataract surgery [5].

DED after cataract surgery has a high prevalence. It affects up to approximately 27% of individuals, including those who do not have dry eye symptoms. It has been reported to last for 1–12 months [6,7], and it damages the corneal and conjunctival epithelium. It can also reduce the number of goblet cells. This is due to repeated drying and washing, disinfectants and drugs, phototoxicity, and surgical trauma. Nerve cutting due to surgical wounds has also been suggested as a cause of DED [8]. Meibomian gland dysfunction (MGD) emerged as a cause of DED after cataract surgery [9,10].

The 0.1% Cyclosporine A cationic emulsion (CsA-CE, Ikervis^®^, Santen Pharmaceutical Co., Ltd., Osaka, Japan) encapsulates the hydrophobic drug cyclosporine A (CsA) with an oil-in-water cationic emulsion (CE), allowing it to remain on the negatively charged ocular surface for an extended period [11]. It improves the symptoms of DED by controlling inflammation on the ocular surface and improving tissue damage. It is widely used for its effectiveness and safety in treating various types of ocular surface diseases and DED [11,12,13]. In addition to the anti-inflammatory effects of CsA, it is thought that the instillation of a CE, which is composed of lipids, can prevent lipid layer damage after cataract surgery [14]. However, no studies have reported the effectiveness of 0.1% CsA-CE for treating DED after cataract surgery. Therefore, we aimed to determine the effectiveness of CsA-CE for treating DED after cataract surgery using an objective evaluation device.

## 2. Materials and Methods

We conducted a retrospective review of the medical records of patients who underwent uncomplicated and straightforward cataract surgery at Kangwon National University Hospital from February 2023 to October 2023. The present study received official approval from the Institutional Review Board of Kangwon National University Hospital, with the designated approval number 2023-08-022. This research was conducted in strict adherence to the fundamental ethical principles outlined in the Declaration of Helsinki by ensuring that all protocols and procedures adhered to the internationally recognized ethical standards for medical research involving human participants. Written informed consent was obtained from all participants to ensure their adequate understanding of this study and voluntary participation before surgery and any subsequent examinations.

The exclusion criteria for this study were carefully defined to ensure the integrity and reliability of the dataset. Patients were excluded if they met any of the following conditions: (a) being younger than 20 years or older than 85 years, as extreme age groups could introduce confounding variables that affect the ocular surface health, cooperation during exams, and surgical outcomes; (b) the presence of ocular diseases that could influence the study outcomes, including but not limited to keratitis, epithelial basement membrane disorder, glaucoma, and retinal diseases, as these conditions could independently affect the ocular surface and tear film stability; (c) a documented history of receiving treatment for DED or related conditions, including medication, within the 4 weeks preceding cataract surgery since recent treatment can mask the natural progression and severity of the condition; (d) use of oral medications or glaucoma eye drops known to influence DED, as systemic and topical medications can alter the tear film composition and confound the results; (e) a history of prior ocular surgeries, such as myopia correction surgery, glaucoma surgery, or retinal surgery, which could introduce variability due to structural and physiological changes in the eye; and (f) intraoperative complications during cataract surgery, including a posterior capsule rupture or any other significant surgical complication, as such events can lead to additional postoperative inflammation and healing responses that may interfere with the study outcomes.

For patients who underwent bilateral cataract surgery, only one eye was included in this study to prevent potential biases associated with inter-eye variability. This careful selection process ensured that the dataset was as controlled and reliable as possible for meaningful analysis and interpretation. Cases with missing records during regular follow-up observations were excluded from this study.

### 2.1. Clinical Examination

**Figure 1:**
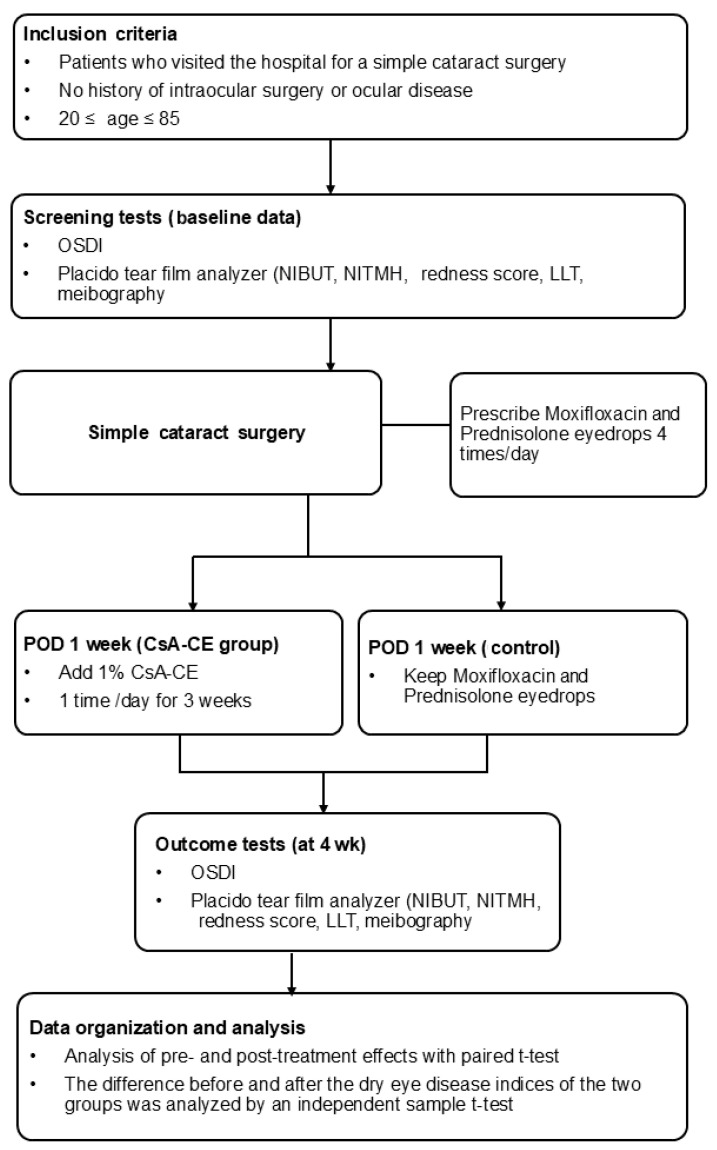
Flowchart of the assessments, treatments, and monitoring of the participants. CsA-CE, 0.1% cyclosporine A cationic emulsion; LLT, lipid layer thickness; NIBUT, non-invasive tear break-up time; NITMH, non-invasive tear meniscus height; OSDI, ocular surface disease index; POD, postoperative day.

All patients who underwent cataract surgery at our medical institution underwent pre- and postoperative comprehensive dry eye evaluations as part of the standardized protocol of the institution. The overall workflow of this evaluation process is illustrated in Figure 1 to provide an overview of the methodology employed in this study.

A thorough assessment of dry eye symptoms was conducted during the initial preoperative visit of the patient to the clinic. All patients were required to complete a standardized and validated questionnaire designed to evaluate subjective dry eye symptoms. The ocular surface disease index (OSDI) questionnaire was administered to quantify the severity and impact of dry eye symptoms experienced by each patient before undergoing cataract surgery. The OSDI score provides an objective measure of patient-reported symptoms, enabling a structured and quantifiable assessment of dry eye severity in a clinical setting.

In addition to the subjective symptom evaluation, an extensive set of objective dry eye parameters was measured using advanced ophthalmic imaging technology. Multiple objective indices related to tear film stability and ocular surface health were assessed using a Placido disk-based tear film analyzer (Keratograph 5M^®^, Oculus, Wetzlar, Germany). The objective parameters evaluated included the non-invasive tear meniscus height (NITMH), which reflects the volume of a tear film at the lower eyelid margin, and the non-invasive tear break-up time (NIBUT), which is used to assess the stability and integrity of the tear film by determining the duration before a tear film rupture occurs.

Furthermore, the ocular redness score was used to quantify conjunctival hyperemia, an indicator of ocular surface inflammation that may be associated with DED. Additionally, the lipid layer thickness (LLT) was evaluated to assess the quality and functionality of the tear film lipid layer, which plays a crucial role in preventing excessive tear evaporation. Finally, meibography was performed to examine the morphology and structural integrity of the meibomian glands, which are essential for maintaining a stable tear film through lipid secretion.

By systematically integrating both subjective symptom evaluation and objective diagnostic testing, our assessment afforded a robust and multidimensional characterization of the dry eye status of patients that underwent cataract surgery. This ensured a thorough understanding of its associated ocular surface changes.

The LLT was approximated by capturing an image at 0.5 s after two natural blinks. The color of each pixel (red, green, and blue scales) was referenced from a lookup table, and the principle of the nearest Euclidean distance was used [15,16]. A more detailed explanation of the lipid layer image analysis is provided in the following section. The NIBUT was measured by inducing the eyes to remain open in their natural state for the longest time. Meibographic images were analyzed using the ImageJ software (version 1.54, National Institutes of Health, Bethesda, MD, USA), and the percentage of viable meibomian gland areas was calculated.

Cataract surgery was performed through a 2.8 mm limbal incision by a single experienced surgeon (Y.L.). The postoperative follow-up was conducted according to the routine protocol of our institution. All patients were administered 0.5% moxifloxacin and 1% prednisolone acetate eye drops four times each for 1 month. The patients were classified into the CsA-CE and control groups based on whether they used 0.1% CsA-CE (Ikervis^®^, Santen Pharmaceutical Co., Ltd., Osaka, Japan) once a day, starting 1 week after surgery. At the hospital visit at 1 month after cataract surgery, the OSDI, NITMH, NIBUT, redness score, and LLT were measured for the baseline examination.

### 2.2. Image Analysis Using Placido Disk Tear Film Analyzer

The Placido-based tear film analyzer used in this study provides dynamic images of the tear lipid layer rather than quantifying its thickness in absolute numerical values. This presents a limitation in that it does not allow for a direct and quantitative assessment of lipid layer thickness changes. However, the advantage of this approach is that researchers can analyze the images in various ways at their discretion, utilizing different methodologies based on visual interpretation. Fu et al. previously reported that it is possible to quantitatively analyze the thickness of the tear lipid layer by extracting oil layer images [15]. Based on the methodology proposed in the study by Fu et al., the authors of the present study conducted additional image analyses to enhance the accuracy and reliability of the measurements.

**Figure 2:**
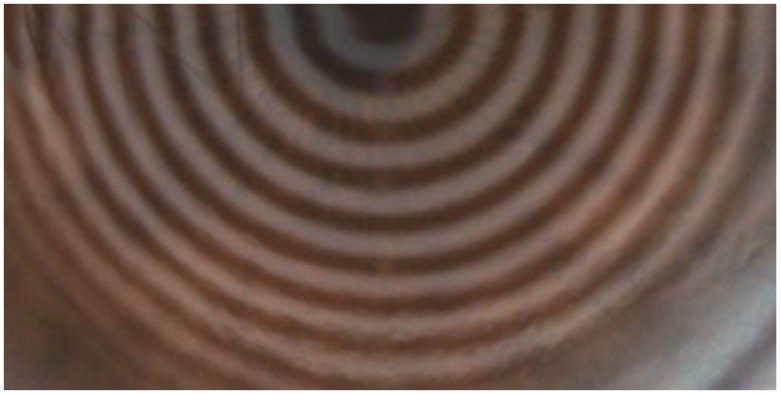
An example of a lipid layer image extracted from a tear film analyzer using Placido rings.

For clinical application, the analysis was performed by capturing an image at exactly 0.5 s after a natural blink from the tear lipid layer video recordings (Figure 2). To ensure the reliability and accuracy of the examination, the upper half of the cornea was excluded, and only the lower half of the cornea was analyzed. This exclusion was necessary because the upper corneal region is often obscured by eyelashes and eyelids, leading to inconsistencies in the measurement and a reduction in the reproducibility of the analysis.

The extracted images were analyzed based on the color of each pixel. To estimate the LLT, the Euclidean distance principle was applied using a look-up table as a reference. The calculation was performed using the following equation:

$$d=\sqrt{({r-R)}^{2}+{\left(g-G\right)}^{2}+{\left(b-B\right)}^{2}}$$

where the lowercase letters (*r*, *g*, *b*) represent the color values of individual pixels from the captured image, while the uppercase letters (*R*, *G*, *B*) represent reference points within the look-up table. The segmentation process was omitted to improve the efficiency of the analysis. The LLT was estimated directly from the unsegmented image using a simplified computational approach. The estimated LLT values were multiplied by a factor of 0.6 and subsequently increased by a value of 10 to facilitate its rapid and simplified measurement.

To assess the spatial distribution of the lipid layer, images were further divided into upper and lower sections. This segmentation was performed using Python (version 3.13, Python Software Foundation, Wilmington, DE, USA) and the Pillow library (version 8.4.0, Python Software Foundation, Wilmington, DE, USA). The LLT of each section was independently calculated using the same methodology described above, and the differences in LLT values between the upper and lower sections were analyzed to evaluate variations in the lipid layer distribution [17].

### 2.3. Statistical Analysis

The normality of the data samples was assessed using the Kolmogorov–Smirnov test. Paired *t*-tests were used to compare the results of all tests before and after cataract surgery. An independent *t*-test was used to compare the dry eye symptoms of the treatment and control groups before and after surgery. Quantitative statistical analyses were performed using SPSS software (version 26.0; IBM Corp., Armonk, NY, USA). Statistical significance was set at *p* < 0.05.

## 3. Results

Among the 253 participants initially reviewed for this retrospective study, 185 eyes from 185 individual patients who met the predefined inclusion criteria were ultimately included in the final analysis. Specifically, 94 eyes from 94 patients were allocated to the CsA-CE group, while the remaining 91 eyes from 91 patients were allocated to the control group.

The mean age of the participants in the CsA-CE group was 70.01 ± 9.09 years, whereas that of the control group was slightly lower at 68.59 ± 10.44 years. However, statistical analysis confirmed that the difference was not significant (*p* = 0.395). The proportion of male participants in the CsA-CE group was 44.7% but slightly higher for the control group at 49.0%. However, this difference was also not statistically significant (*p* = 0.727), suggesting that the gender ratios of the two groups were comparable. The preoperative visual acuity, which was measured using the logarithm of the minimum angle of resolution (LogMAR VA), was found to be 0.43 ± 0.43 for the CsA-CE group and 0.56 ± 0.64 for the control group. The statistical analysis demonstrated that this difference was not significant (*p* = 0.197). The spherical equivalent (SE) values were 0.03 ± 2.91 diopters (D) for the CsA-CE group and −0.16 ± 3.71 D for the control group, with no statistically significant difference between the two groups (*p* = 0.737). In terms of the corneal curvature, the mean keratometry (K mean) values were 44.48 ± 1.53 D for the CsA-CE group and 44.50 ± 1.59 D for the control group. The difference between the two groups was statistically insignificant (*p* = 0.950). The keratometric astigmatism (K astig) values were nearly identical for the two groups at 1.03 ± 0.85 D for the CsA-CE group and 1.01 ± 0.70 D for the control group, with no significant difference observed (*p* = 0.915). The anterior chamber depth was measured at 3.11 ± 0.42 mm for the CsA-CE group and 3.09 ± 0.48 mm for the control group, showing no significant difference (*p* = 0.775). The axial length (AXL) values were 23.64 ± 1.01 mm for the CsA-CE group and 23.35 ± 1.09 mm for the control group, with no statistically significant variation between the groups (*p* = 0.119).

Regarding the cataract severity, the cortical (C), nuclear (N), and posterior subcapsular (*p*) cataract grades were assessed. The mean cortical cataract grade was 2.42 ± 1.43 for the CsA-CE group and 2.38 ± 1.62 for the control group, showing no significant difference (*p* = 0.876). The nuclear cataract grade was 3.17 ± 0.99 for the CsA-CE group and 3.42 ± 1.14 for the control group, with no significant difference (*p* = 0.169). The posterior subcapsular cataract grade was 1.87 ± 1.94 for the CsA-CE group and 1.78 ± 1.97 for the control group, with no significant difference (*p* = 0.784). The mean ECC value was 2685.19 ± 289.51 cells/mm² for the CsA-CE group and 2685.34 ± 335.50 cells/mm² for the control group. These values showed no statistically significant difference (*p* = 0.998). The central corneal thickness (CCT) was 535.82 ± 36.96 µm for the CsA-CE group and 538.00 ± 38.54 µm for the control group, with no significant difference (*p* = 0.791).

Overall, the statistical comparisons demonstrated no significant differences between the CsA-CE and control groups in terms of the demographic characteristics and general ocular parameters. The detailed demographic and clinical characteristics are systematically summarized in Table 1.

In the preoperative DED examinations, multiple clinical parameters, including the OSDI, NITMH, NIBUT, conjunctival redness scores, and LLT, were measured, and meibography was performed. The statistical analysis showed no significant differences in any of these parameters between the CsA-CE group and the control group.

The OSDI was 19.26 ± 20.19 for the CsA-CE group and 17.40 ± 22.78 for the control group (*p* = 0.614). The NITMH was 0.32 ± 0.19 mm for the CsA-CE group and 0.31 ± 0.15 mm for the control group (*p* = 0.731). For the tear film stability, the first NIBUT was 7.46 ± 4.05 s for the CsA-CE group and 8.96 ± 5.14 s for the control group (*p* = 0.070). The mean NIBUT was 10.97 ± 4.08 s for the CsA-CE group and 11.71 ± 4.50 s for the control group (*p* = 0.314). The conjunctival redness scores were assessed in different regions. The bulbar temporal score was 1.64 ± 0.61 for the CsA-CE group and 1.81 ± 0.69 for the control group (*p* = 0.122). The limbal temporal score was 1.25 ± 0.51 for the CsA-CE group and 1.31 ± 0.60 for the control group (*p* = 0.519). The bulbar nasal score was 1.63 ± 1.16 for the CsA-CE group and 1.49 ± 0.71 for the control group (*p* = 0.414). The limbal nasal score was 1.12 ± 0.54 in both groups (*p* = 0.964). The total limbal score was 1.58 ± 0.54 for the CsA-CE group and 1.69 ± 0.62 for the control group (*p* = 0.250). The LLT was 70.29 ± 23.27 nm for the CsA-CE group and 74.86 ± 28.54 nm for the control group (*p* = 0.300). Meibography showed viable meibomian gland areas of 58.62 ± 9.51% for the CsA-CE group and 58.11 ± 8.94% for the control group (*p* = 0.755).

Overall, no statistically significant differences were found between the two groups for any of the preoperative DED parameters, confirming comparable baseline ocular surface conditions.

Table 2 presents the postoperative changes in the DED symptom scores and indices at 1 month after cataract surgery for both the CsA-CE and control groups.

The OSDI significantly decreased for the CsA-CE group from 19.26 ± 20.19 preoperatively to 14.58 ± 12.50 at 1 month postoperation (*p* = 0.046), indicating an improvement in subjective dry eye symptoms. In contrast, no statistically significant change was observed in the control group, with OSDI values of 17.40 ± 22.78 preoperatively and 19.20 ± 19.41 postoperatively (*p* = 0.654).

The NITMH showed no statistically significant change for either group. The preoperative and postoperative NITMHs were 0.32 ± 0.19 mm and 0.32 ± 0.17 mm for the CsA-CE group (*p* = 0.853) and 0.31 ± 0.15 mm and 0.32 ± 0.17 mm for the control group (*p* = 0.602), respectively.

The first NIBUT did not show a statistically significant change for either group. It was 7.46 ± 4.05 s preoperatively and 8.54 ± 5.02 s postoperatively (*p* = 0.087) for the CsA-CE group. The control group showed a decrease from 8.96 ± 5.14 s preoperatively to 7.46 ± 5.21 s postoperatively (*p* = 0.189), but this change was not statistically significant. On the other hand, the average NIBUT significantly increased for the CsA-CE group from 10.97 ± 4.08 s preoperatively to 13.00 ± 4.96 s postoperatively (*p* = 0.002). However, no statistically significant change was observed for the control group, with pre- and postoperative values of 11.71 ± 4.50 s and 11.55 ± 5.18 s, respectively (*p* = 0.876).

The conjunctival redness increased postoperatively in certain regions. For the CsA-CE group, the limbal nasal redness score increased significantly from 1.12 ± 0.54 to 1.30 ± 0.59 (*p* = 0.005), and the total limbal redness score increased significantly from 1.58 ± 0.54 to 1.73 ± 0.59 (*p* = 0.017). For the control group, the conjunctival redness scores significantly increased from 1.49 ± 0.71 to 1.88 ± 0.63 for the bulbar nasal (*p* < 0.001), 1.12 ± 0.69 to 1.50 ± 0.73 for the limbal nasal (*p* = 0.001), and 1.69 ± 0.62 to 1.96 ± 0.56 for the total limbal (*p* = 0.012).

The LLT significantly increased for the CsA-CE group from 70.29 ± 23.27 nm preoperatively to 86.41 ± 21.89 nm postoperatively (*p* < 0.001). In contrast, no statistically significant change was observed for the control group, with preoperative and postoperative values of 74.86 ± 28.54 nm and 78.22 ± 26.59 nm, respectively (*p* = 0.519).

The vertical difference in the LLT did not show a statistically significant change for either group. For the CsA-CE group, the preoperative and postoperative values were 18.33 ± 8.49 nm and 17.80 ± 9.57 nm, respectively (*p* = 0.536). For the control group, the values were 17.84 ± 6.84 nm and 17.54 ± 8.28 nm, respectively (*p* = 0.786).

The meibography results showed no statistically significant changes for the viable meibomian gland area for either group. For the CsA-CE group, the values were 58.62 ± 9.51% preoperatively and 58.69 ± 8.39% postoperatively (*p* = 0.167). For the control group, the preoperative and postoperative values were 58.11 ± 8.94% and 57.74 ± 7.08%, respectively (*p* = 0.799).

Overall, significant improvements were observed for the OSDI, average NIBUT, and LLT for the CsA-CE group. For the control group, the conjunctival redness score increased but no statistically significant changes in other parameters were observed. The detailed findings are summarized in Table 2.

**Figure 3:**
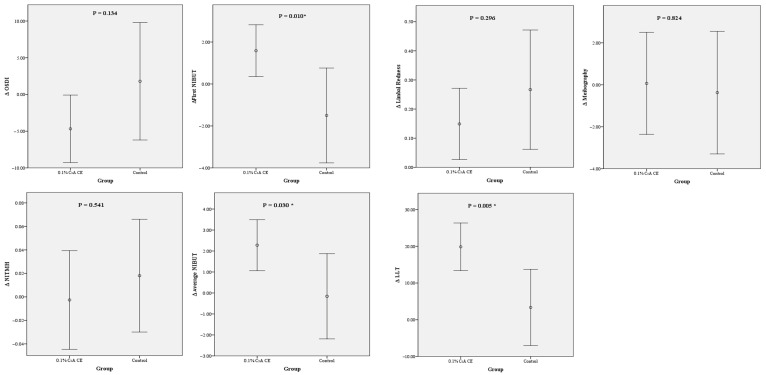
Differences between the dry eye disease parameter values obtained pre- and postoperatively (after–before) for the 0.1% cyclosporine A cationic emulsion (0.1% CsA-CE) and control groups. The NIBUT and LLT changes in the two groups were significantly different (independent *t*-test). Changes in the OSDI, NITMH, First NIBUT, Average NIBUT, Limbal redness, LLT, and Meibography. The first NIBUT, average NIBUT, and LLT were significantly higher for the CsA-CE group than for the control group. LLT, lipid layer thickness; NIBUT, non-invasive tear break-up time; NITMH, non-invasive tear meniscus height; OSDI, ocular surface disease index. * Statistically significant.

A comparison of the changes in the preoperative and postoperative values of the DED parameters revealed significantly greater NIBUT and LLT changes for the CsA-CE group than for the control group. The statistical analysis demonstrated that the increases in both the NIBUT and LLT at 1 month after cataract surgery were significantly larger for the CsA-CE group, indicating a notable improvement in these tear film stability markers. These findings are represented in Figure 3.

No side effects, such as toxic keratitis, infective keratitis, or hypersensitivity reactions, were observed after using the drug. Continuous monitoring further verified the absence of unexpected side effects, and no participants required discontinuation due to safety concerns.

## 4. Discussion

DED negatively impacts patient satisfaction after cataract surgery [5]. It may occur postoperatively in 37.4% of patients without existing DED, which can reduce the quality of vision and cause patient discomfort. Therefore, thorough and effective DED evaluation and treatment before and after cataract surgery are required [6,10].

DED after cataract surgery is associated with decreased tear secretion and break-up time [18]. Increased concentrations of inflammatory mediators may also contribute to this condition [19]. MGD, or a decrease in the tear lipid layer after cataract surgery, is also a major cause of DED [20]. The efficacies of several dry eye medications for treating DED after cataract surgery have been reported. Previous studies reported that sodium hyaluronate, diquafosol, and rebamipide help control DED after cataract surgery [21,22,23].

The results of this study suggest that 0.1% CsA-CE may be effective at controlling DED after cataract surgery. The therapeutic effects of cyclosporine on DED have been widely reported. A study reported improved corneal staining scores of patients with moderate-to-severe dry eye, as well as the Schirmer test and OSDI scores through once-daily administration [12,24,25]. In this study, the group treated with Cyclosporine showed a statistically significant change in the OSDI, which decreased from 19.26 to 14.58 and fell slightly short of the minimal clinically important difference but remained meaningful [26].

Several studies reported various therapeutic effects of cyclosporine eyedrops, although their findings have not been consistent. They particularly highlighted the increase in goblet cells, which are believed to play a crucial role in the management of dry eye [27,28]. DED directly or indirectly damages the ocular surface via a persistent inflammatory chain reaction. Cyclosporine reduces inflammation by targeting specific inflammatory pathways to break this inflammatory cycle [11,29].

Repeated drying with irrigation, drug toxicity, surface damage, and phototoxicity that occur during cataract surgery are causes of postoperative DED [8]. CsA-CE is expected to have a therapeutic effect on DED that occurs after cataract surgery, similar to other dry eye treatments. The results of this study showed that the postoperative OSDI was lower for the CsA-CE group, and the average NIBUT improved for the CsA-CE group (Table 2). The key to the additional therapeutic effects of CsA is the cationic oil-in-water emulsion surrounding the CsA component. To extend the residence time of cyclosporine on the ocular surface, a cyclic polypeptide with low water solubility and positively charged oil nanodroplets stabilized the tear film and increased the lipid layer, providing moisturizing and lubricating effects [14,30,31]. This study was the first to report that the thickness of the tear lipid layer may increase in eyes treated with CsA-CE. This suggests that the CE coating effect can prevent dry eye caused by MGD, which is one of the main causes of DED after cataract surgery [9,10,20].

The relationship between the spatial distribution of the tear film lipid layer and the pathophysiology of DED remains incompletely understood and has not yet been fully elucidated. While an increase in the absolute thickness of the tear lipid layer is often considered beneficial for ocular surface health, it is equally important to recognize that the uniformity and distribution of the lipid layer across the corneal surface may play a crucial role in maintaining tear film stability [32]. An uneven or irregular distribution of the lipid layer can contribute to localized tear film instability, leading to excessive evaporation in certain areas and exacerbating dry eye symptoms. Beyond simply increasing the thickness of the lipid layer, achieving a well-balanced and evenly distributed lipid layer may be essential for the effective management of DED [33].

A previous study that employed the same imaging device used in the present study reported that the vertical difference in LLT significantly decreased following intensive pulsed light (IPL) therapy. This indicates that IPL may contribute to a more homogenous lipid layer distribution [17]. This finding underscores the potential role of lipid layer uniformity, in addition to the overall thickness, in alleviating symptoms associated with DED. IPL treatment is known to directly stimulate meibomian gland function, facilitating the secretion of meibum and enhancing both the quantity and distribution of the lipid layer.

In contrast, the results of our present study show that there was no statistically significant improvement in the vertical distribution uniformity of the lipid layer after the treatment, although the overall thickness of the tear lipid layer significantly increased. This discrepancy in findings may be attributed to the fundamentally different mechanisms of action of the two treatment modalities examined. IPL therapy directly targets the meibomian glands, enhancing their ability to secrete lipids and promoting a more uniform lipid layer across the corneal surface [34]. Conversely, CsA-CE exerts its therapeutic effects through direct application to the ocular surface, reducing inflammation and increasing the lipid layer thickness without directly modulating the meibomian gland function [35]. As a result, CsA-CE does not necessarily enhance the uniformity of lipid distribution in the same manner as IPL, although it contributes to an increase in the average LLT.

In the present study, both the treatment and control groups received postoperative steroid therapy, which comprised the use of potent anti-inflammatory agents widely used in clinical practice to manage inflammation following ocular surgery. Previous studies reported that combination therapy with cyclosporine and steroids may be beneficial for severe DED [36]. However, our study did not include patients with severe DED, and we cannot be certain about the additional effects of steroid use. Given this common use of steroids in both groups, it is reasonable to infer that the therapeutic effects of CsA-CE observed in this study may be primarily attributable to the coating and stabilizing effects of the CE rather than solely to its anti-inflammatory properties. The CE formulation is designed to enhance retention on the ocular surface, which improves the lipid layer stability and provides additional protection to the tear film. This may have contributed to the observed improvements in DED parameters following cataract surgery [37].

One of the notable limitations of this study was the relatively short follow-up. As previous research suggested, DED symptoms following cataract surgery can persist for more than 6 months postoperation. Therefore, a long-term observational study with an extended follow-up would be necessary to fully assess the prolonged effects of CsA-CE in this patient population. Conducting an extended longitudinal study would allow for a more comprehensive evaluation of the sustained impact of CsA-CE on postoperative ocular surface health and tear film stability over time. Additionally, patients in both groups were prescribed a combination of antibiotics and steroid eye drops postoperatively due to the standard clinical protocol for cataract surgery. This adjunctive treatment may have had a confounding effect, potentially masking or interfering with the isolated effects of CsA-CE on ocular surface health. Therefore, future research should consider study designs that better isolate the effects of CsA-CE by minimizing or controlling the influence of other medications, although this study provided valuable insights. Another limitation of this study was that certain conventional dry eye diagnostic parameters, such as the ocular surface staining score and the Schirmer test, were not included in the analysis. These tests are commonly used to assess DED severity by measuring the tear production and corneal epithelial damage. Additionally, the LLT was not directly measured but rather inferred through image analysis. In future studies, it will be necessary to analyze how LLT measurements obtained through this method correlate with traditional dry eye parameters. Conjunctival redness is not yet a fully validated indicator of DED. However, efforts were made to enhance the reliability and objectivity of the findings by focusing on instrument-based, quantitative dry eye assessments rather than subjective scoring methods, given the unblinded nature of this study. Specifically, dry eye parameters were assessed using specialized imaging equipment operated by a skilled and experienced examiner, minimizing the potential for bias introduced by subjective interpretation. Despite these efforts, a double-blind, randomized controlled trial should be conducted in the future to eliminate any remaining bias and strengthen the evidence supporting the use of CsA-CE in postoperative DED management to further improve the validity and reproducibility of the findings.

## 5. Conclusions

In conclusion, the findings of this study suggest that CsA-CE following cataract surgery is effective and safe for both the prevention and management of DED. Given its beneficial effects on tear film stability, lipid layer enhancement, and ocular surface protection, CsA-CE is a promising adjunct therapy that may help mitigate the risk of postoperative dry eye symptoms and improve patient comfort and visual outcomes. However, further well-designed, long-term studies with larger sample sizes and double-blind methodologies are required to comprehensively evaluate its long-term therapeutic benefits and establish it as a standard treatment option for postoperative DED.

## Figures and Tables

**Table 1:** Comparison of the demographic data and dry eye indices measured using the Placido disk tear film analyzer before the cataract surgeries in the cyclosporine and control groups.

	CsA-CE Group (*n* = 94)	Control Group (*n* = 91)	*p*-Value *
Age (years)	70.01 ± 9.09	68.59 ± 10.44	0.395
Sex (male)	44.7%	49%	0.727
LogMAR VA	0.43 ± 0.43	0.56 ± 0.64	0.197
SE (D)	0.03 ± 2.91	−0.16 ± 3.71	0.737
K mean (D)	44.48 ± 1.53	44.50 ± 1.59	0.950
K astig (D)	1.03 ± 0.85	1.01 ± 0.70	0.915
ACD (mm)	3.11 ± 0.42	3.09 ± 0.48	0.775
AXL (mm)	23.64 ± 1.01	23.35 ± 1.09	0.119
C	2.42 ± 1.43	2.38 ± 1.62	0.876
N	3.17 ± 0.99	3.42 ± 1.14	0.169
P	1.87 ± 1.94	1.78 ± 1.97	0.784
ECC (cells/mm^2^)	2685.19 ± 289.51	2685.34 ± 335.50	0.998
CCT (μm)	535.82 ± 36.96	538 ± 38.54	0.791
OSDI	19.26 ± 20.19	17.40 ± 22.78	0.614
NITMH (mm)	0.32 ± 0.19	0.31 ± 0.0.15	0.731
NIBUT first (s)	7.46 ± 4.05	8.96 ± 5.14	0.070
NIBUT average (s)	10.97 ± 4.08	11.71 ± 4.50	0.314
Conjunctival redness			
Bulbar temporal	1.64 ± 0.61	1.81 ± 0.69	0.122
Limbal temporal	1.25 ± 0.51	1.31 ± 0.60	0.519
Bulbar nasal	1.63 ± 1.16	1.49 ± 0.71	0.414
Limbal nasal	1.12 ± 0.54	1.12 ± 0.69	0.964
Limbal total	1.58 ± 0.54	1.69 ± 0.62	0.250
LLT (nm)	70.29 ± 23.27	74.86 ± 28.54	0.300
Meibography (%)	58.62 ± 9.51	58.11 ± 8.94	0.755

Values are presented as mean ± standard deviation. ACD, anterior chamber depth; AXL, axial length; C, cortical spoking cataract grade; CCT, central corneal thickness; CsA-CE, cyclosporine A cationic emulsion; ECC, endothelial cell count; K, mean keratometry; K astig, steep keratometry; LLT, lipid layer thickness; LogMAR VA, logarithm of minimal angle of resolution visual acuity; N, nuclear sclerotic cataract grade; NIBUT, non-invasive tear break-up time; NITMH, non-invasive tear meniscus height; OSDI, ocular surface disease index; P, posterior subcapsular cataract grade; SE, spherical equivalent. * Independent *t*-test.

**Table 2:** Dry eye disease symptom score and indices measured using the Placido disk tear film analyzer 1 month after cataract surgery for both groups.

Parameters	CsA-CE Group (*n* = 94)			Control Group (*n* = 91)		
	Pre	Post-1M	*p*-Value *	Pre	Post-1M	*p*-Value *
OSDI	19.26 ± 20.19	14.58 ± 12.50	0.046	17.40 ± 22.78	19.20 ± 19.41	0.654
NITMH (mm)	0.32 ± 0.19	0.32 ± 0.17	0.853	0.31 ± 0.0.15	0.32 ± 0.17	0.602
NIBUT first (s)	7.46 ± 4.05	8.54 ± 5.02	0.087	8.96 ± 5.14	7.46 ± 5.21	0.189
NIBUT average (s)	10.97 ± 4.08	13.00 ± 4.96	0.002	11.71 ± 4.50	11.55 ± 5.18	0.876
Conjunctival redness						
Bulbar temporal	1.64 ± 0.61	1.75 ± 0.60	0.107	1.81 ± 0.69	1.97 ± 0.62	0.148
Limbal temporal	1.25 ± 0.51	1.35 ± 0.60	0.122	1.31 ± 0.60	1.48 ± 0.67	0.091
Bulbar nasal	1.63 ± 1.16	1.81 ± 0.66	0.170	1.49 ± 0.71	1.88 ± 0.63	0.000
Limbal nasal	1.12 ± 0.54	1.30 ± 0.59	0.005	1.12 ± 0.69	1.50 ± 0.73	0.001
Limbal total	1.58 ± 0.54	1.73 ± 0.59	0.017	1.69 ± 0.62	1.96 ± 0.56	0.012
LLT (nm)	70.29 ± 23.27	86.41 ± 21.89	<0.001	74.86 ± 28.54	78.22 ± 26.59	0.519
Vertical difference (nm)	18.33 ±8.49	17.80 ± 9.57	0.536	17.84 ± 6.84	17.54 ± 8.28	0.786
Meibography(%)	58.62 ± 9.51	58.69 ± 8.39	0.167	58.11 ± 8.94	57.74 ± 7.08	0.799

Values are presented as mean ± standard deviation. CsA-CE, cyclosporine A cationic emulsion; LLT, lipid layer thickness; NIBUT, non-invasive tear break-up time; NITMH, non-invasive tear meniscus height; OSDI, ocular surface disease index. * Independent *t*-test.

## Data Availability

The datasets generated during and/or analyzed during the current study are available from the corresponding author upon reasonable request.

## Abbreviations

The following abbreviations are used in this manuscript:

CE	Cationic emulsion
CsA	Cyclosporine A cationic
DED	Dry eye disease
IPL	Intense pulsed light
LLT	Lipid layer thickness
MGD	Meibomian gland dysfunction
NIBUT	Non-invasive tear break-up time
NITMH	Non-invasive tear meniscus height
OSDI	Ocular surface disease index

## References

[1] (2000). Vision 2020: The cataract challenge. Community Eye Health.

[2] Pascolini D., Mariotti S.P. (2012). Global estimates of visual impairment: 2010. Br. J. Ophthalmol..

[3] Hardten D.R. (2008). Dry eye disease in patients after cataract surgery. Cornea.

[4] Li X.M., Hu L., Hu J., Wang W. (2007). Investigation of dry eye disease and analysis of the pathogenic factors in patients after cataract surgery. Cornea.

[5] Gibbons A., Ali T.K., Waren D.P., Donaldson K.E. (2016). Causes and correction of dissatisfaction after implantation of presbyopia-correcting intraocular lenses. Clin. Ophthalmol..

[6] Miura M., Inomata T., Nakamura M., Sung J., Nagino K., Midorikawa-Inomata A., Zhu J., Fujimoto K., Okumura Y., Fujio K. (2022). Prevalence and Characteristics of Dry Eye Disease After Cataract Surgery: A Systematic Review and Meta-Analysis. Ophthalmol. Ther..

[7] Sajnani R., Raia S., Gibbons A., Chang V., Karp C.L., Sarantopoulos C.D., Levitt R.C., Galor A. (2018). Epidemiology of Persistent Postsurgical Pain Manifesting as Dry Eye-Like Symptoms After Cataract Surgery. Cornea.

[8] Naderi K., Gormley J., O’Brart D. (2020). Cataract surgery and dry eye disease: A review. Eur. J. Ophthalmol..

[9] Qiu J.J., Sun T., Fu S.H., Yu Y.F., You Z.P., Zhang Q., Liu F., Huang J.Q., Wang Z.H. (2020). A study of dry eye after cataract surgery in MGD patients. Int. Ophthalmol..

[10] Sutu C., Fukuoka H., Afshari N.A. (2016). Mechanisms and management of dry eye in cataract surgery patients. Curr. Opin. Ophthalmol..

[11] Hoy S.M. (2017). Ciclosporin Ophthalmic Emulsion 0.1%: A Review in Severe Dry Eye Disease. Drugs.

[12] Baudouin C., de la Maza M.S., Amrane M., Garrigue J.S., Ismail D., Figueiredo F.C., Leonardi A. (2017). One-Year Efficacy and Safety of 0.1% Cyclosporine a Cationic Emulsion in the Treatment of Severe Dry Eye Disease. Eur. J. Ophthalmol..

[13] Geerling G., Hamada S., Trocme S., Raeder S., Chen X., Fassari C., Lanzl I., The PERSPECTIVE Study Group (2022). Real-World Effectiveness, Tolerability and Safety of Cyclosporine A 0.1% Cationic Emulsion in Severe Keratitis and Dry Eye Treatment. Ophthalmol. Ther..

[14] Daull P., Amrane M., Ismail D., Georgiev G., Cwiklik L., Baudouin C., Leonardi A., Garhofer G., Garrigue J.S. (2020). Cationic Emulsion-Based Artificial Tears as a Mimic of Functional Healthy Tear Film for Restoration of Ocular Surface Homeostasis in Dry Eye Disease. J. Ocul. Pharmacol. Ther..

[15] Fu P.I., Fang P.C., Ho R.W., Chao T.L., Cho W.H., Lai H.Y., Hsiao Y.T., Kuo M.T. (2020). Determination of Tear Lipid Film Thickness Based on a Reflected Placido Disk Tear Film Analyzer. Diagnostics.

[16] Hwang H., Jeon H.J., Yow K.C., Hwang H.S., Chung E. (2017). Image-based quantitative analysis of tear film lipid layer thickness for meibomian gland evaluation. Biomed. Eng. Online.

[17] Lee Y., Lee S.W., Yun J.K., Han S.Y., Choi C.Y. (2025). Changes in the distribution of the tear film lipid layer after intensive pulsed light combined with meibomian gland expression in patients with meibomian gland dysfunction. PLoS ONE.

[18] Choi Y.J., Park S.Y., Jun I., Choi M., Seo K.Y., Kim E.K., Kim T.I. (2018). Perioperative Ocular Parameters Associated With Persistent Dry Eye Symptoms After Cataract Surgery. Cornea.

[19] Park Y., Hwang H.B., Kim H.S. (2016). Observation of Influence of Cataract Surgery on the Ocular Surface. PLoS ONE.

[20] Han K.E., Yoon S.C., Ahn J.M., Nam S.M., Stulting R.D., Kim E.K., Seo K.Y. (2014). Evaluation of dry eye and meibomian gland dysfunction after cataract surgery. Am. J. Ophthalmol..

[21] Cui L., Li Y., Lee H.S., Yang J.M., Choi W., Yoon K.C. (2018). Effect of diquafosol tetrasodium 3% on the conjunctival surface and clinical findings after cataract surgery in patients with dry eye. Int. Ophthalmol..

[22] Jee D., Park M., Lee H.J., Kim M.S., Kim E.C. (2015). Comparison of treatment with preservative-free versus preserved sodium hyaluronate 0.1% and fluorometholone 0.1% eyedrops after cataract surgery in patients with preexisting dry-eye syndrome. J. Cataract. Refract. Surg..

[23] Kato K., Miyake K., Kondo N., Asano S., Takeda J., Takahashi A., Takashima Y., Kondo M. (2017). Conjunctival Goblet Cell Density Following Cataract Surgery With Diclofenac Versus Diclofenac and Rebamipide: A Randomized Trial. Am. J. Ophthalmol..

[24] Baudouin C., Figueiredo F.C., Messmer E.M., Ismail D., Amrane M., Garrigue J.S., Bonini S., Leonardi A. (2017). A randomized study of the efficacy and safety of 0.1% cyclosporine A cationic emulsion in treatment of moderate to severe dry eye. Eur. J. Ophthalmol..

[25] Leonardi A., Van Setten G., Amrane M., Ismail D., Garrigue J.S., Figueiredo F.C., Baudouin C. (2016). Efficacy and safety of 0.1% cyclosporine A cationic emulsion in the treatment of severe dry eye disease: A multicenter randomized trial. Eur. J. Ophthalmol..

[26] Miller K.L., Walt J.G., Mink D.R., Satram-Hoang S., Wilson S.E., Perry H.D., Asbell P.A., Pflugfelder S.C. (2010). Minimal clinically important difference for the ocular surface disease index. Arch. Ophthalmol..

[27] de Paiva C.S., Pflugfelder S.C., Ng S.M., Akpek E.K. (2019). Topical cyclosporine A therapy for dry eye syndrome. Cochrane Database Syst. Rev..

[28] Kunert K.S., Tisdale A.S., Gipson I.K. (2002). Goblet cell numbers and epithelial proliferation in the conjunctiva of patients with dry eye syndrome treated with cyclosporine. Arch. Ophthalmol..

[29] Baudouin C., Irkec M., Messmer E.M., Benitez-Del-Castillo J.M., Bonini S., Figueiredo F.C., Geerling G., Labetoulle M., Lemp M., Rolando M. (2018). Clinical impact of inflammation in dry eye disease: Proceedings of the ODISSEY group meeting. Acta Ophthalmol..

[30] Daull P., Lallemand F., Garrigue J.S. (2014). Benefits of cetalkonium chloride cationic oil-in-water nanoemulsions for topical ophthalmic drug delivery. J. Pharm. Pharmacol..

[31] Daull P., Lallemand F., Philips B., Lambert G., Buggage R., Garrigue J.S. (2013). Distribution of cyclosporine A in ocular tissues after topical administration of cyclosporine A cationic emulsions to pigmented rabbits. Cornea.

[32] Szczesna-Iskander D.H. (2018). Post-blink tear film dynamics in healthy and dry eyes during spontaneous blinking. Ocul. Surf..

[33] Goto E., Tseng S.C. (2003). Differentiation of lipid tear deficiency dry eye by kinetic analysis of tear interference images. Arch. Ophthalmol..

[34] Gupta A.S., Massaro M., Bunya V.Y. (2024). Intense pulsed light treatment for the management of meibomian gland dysfunction. Curr. Opin. Ophthalmol..

[35] Kang M.S., Shin J., Kwon J.M., Huh J., Lee J.E. (2021). Efficacy of 0.05% cyclosporine A on the lipid layer and meibomian glands after cataract surgery: A randomized, double-masked study. PLoS ONE.

[36] Byun Y.J., Kim T.I., Kwon S.M., Seo K.Y., Kim S.W., Kim E.K., Park W.C. (2012). Efficacy of combined 0.05% cyclosporine and 1% methylprednisolone treatment for chronic dry eye. Cornea.

[37] Labetoulle M., Garhofer G., Ismail D., Garrigue J.S., Amrane M., Guillon M., Aragona P., Baudouin C. (2024). Review of clinical outcomes of a cationic emulsion tear substitute in patients with dry eye disease. Acta Ophthalmol..

